# Development of the livestock pathogen *Trypanosoma (Nannomonas) simiae* in the tsetse fly with description of putative sexual stages from the proboscis

**DOI:** 10.1186/s13071-023-05847-5

**Published:** 2023-07-11

**Authors:** Lori Peacock, Chris Kay, Clare Collett, Mick Bailey, Wendy Gibson

**Affiliations:** 1 School of Biological Sciences, University of Bristol, Bristol, BS8 1TQ UK; 2 Bristol Veterinary School, University of Bristol, Langford, Bristol, BS40 7DU UK; 3 Present Address: Pathogen Immunology Group, UK Health Security Agency, Porton Down, Salisbury, SP4 0JG Wiltshire UK

**Keywords:** *Trypanosoma simiae*, Sexual reproduction, Tsetse fly, Life cycle, Meiosis, Gamete

## Abstract

**Background:**

Tsetse-transmitted African animal trypanosomiasis is recognised as an important disease of ruminant livestock in sub-Saharan Africa, but also affects domestic pigs, with *Trypanosoma simiae* notable as a virulent suid pathogen that can rapidly cause death. *Trypanosoma simiae* is widespread in tsetse-infested regions, but its biology has been little studied compared to *T. brucei* and *T. congolense*.

**Methods:**

*Trypanosoma simiae* procyclics were cultured in vitro and transfected using protocols developed for *T. brucei*. Genetically modified lines, as well as wild-type trypanosomes, were transmitted through tsetse flies, *Glossina pallidipes*, to study *T. simiae* development in the tsetse midgut, proventriculus and proboscis. The development of proventricular trypanosomes was also studied in vitro. Image and mensural data were collected and analysed.

**Results:**

A *PFR1::YFP* line successfully completed development in tsetse, but a *YFP::HOP1* line failed to progress beyond midgut infection. Analysis of image and mensural data confirmed that the vector developmental cycles of *T. simiae* and *T. congolense* are closely similar, but we also found putative sexual stages in *T. simiae*, as judged by morphological similarity to these stages in *T. brucei*. Putative meiotic dividers were abundant among *T. simiae* trypanosomes in the proboscis, characterised by a large posterior nucleus and two anterior kinetoplasts. Putative gametes and other meiotic intermediates were also identified by characteristic morphology. In vitro development of proventricular forms of *T. simiae* followed the pattern previously observed for *T. congolense*: long proventricular trypanosomes rapidly attached to the substrate and shortened markedly before commencing cell division.

**Conclusions:**

To date, *T. brucei* is the only tsetse-transmitted trypanosome with experimentally proven capability to undergo sexual reproduction, which occurs in the fly salivary glands. By analogy, sexual stages of *T. simiae* or *T. congolense* are predicted to occur in the proboscis, where the corresponding portion of the developmental cycle takes place. While no such stages have been observed in *T. congolense*, for *T. simiae* putative sexual stages were abundant in the tsetse proboscis. Although our initial attempt to demonstrate expression of a YFP-tagged, meiosis-specific protein was unsuccessful, the future application of transgenic approaches will facilitate the identification of meiotic stages and hybrids in *T. simiae*.

**Graphical Abstract:**

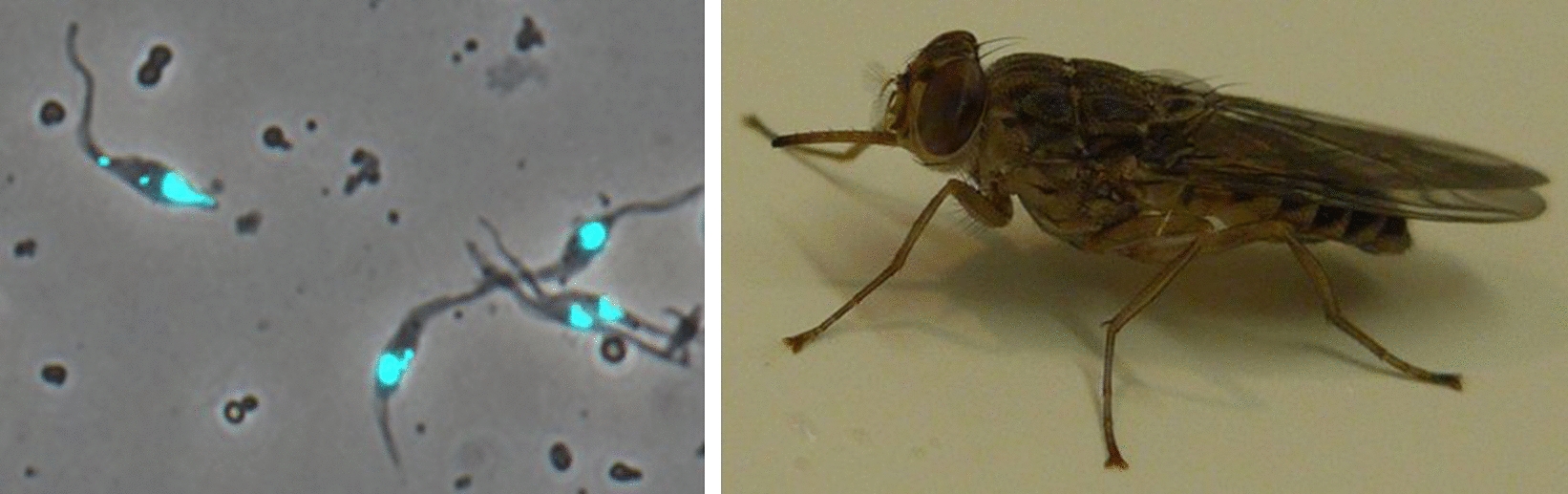

**Supplementary Information:**

The online version contains supplementary material available at 10.1186/s13071-023-05847-5.

## Background

Tsetse-transmitted trypanosomes are notorious pathogens of cattle in sub-Saharan Africa, causing the disease nagana or African animal trypanosomiasis (AAT), but it is often overlooked that these trypanosomes also cause disease of varying severity in a range of other livestock species. Indeed, *Trypanosoma simiae* causes acute porcine trypanosomiasis and was once described as “the lightning destroyer of the domestic pig” because of the swift and fatal outcome of infection [1]. Though best known as a virulent pathogen of domestic pigs, the host range of *T. simiae* encompasses cattle and camels [1–3] and it is frequently found in tsetse across sub-Saharan Africa [4–11].

Inarguably, based on pathogenicity and prevalence, *T. simiae* deserves more attention, but there has been comparatively little recent research on this species compared to its better known relative, *Trypanosoma congolense*. Both species were assigned to subgenus *Nannomonas*, because they follow a similar developmental pathway in the tsetse fly vector [1]. Briefly, bloodstream form trypanosomes differentiate to procyclic forms in the tsetse midgut, multiply and then move anteriorly to colonise first the proventriculus or cardia (the valve between the mid- and foregut) and subsequently the proboscis. Infective metacyclics develop in the hypopharynx, the single duct that carries saliva from the paired salivary glands to the tip of the proboscis, and are inoculated with the saliva as the fly feeds on an animal; the complete cycle of development takes about 20 days [1].

This developmental cycle was described decades ago, before the discovery that trypanosomes may undergo sexual reproduction in the tsetse vector [12] and the description of meiotic dividing cells and gametes [13–15]. While these results pertain to *Trypanosoma brucei*, there is a strong probability that sexual stages might also be found among trypanosomes of subgenus *Nannomonas*, as there is evidence that *T. congolense* undergoes genetic exchange in nature [16, 17]. However, no obvious sexual stages were identified during the developmental cycle of *T. congolense* in tsetse [18], despite subsequent demonstration that hybrids are found in the tsetse proboscis in laboratory crosses (Peacock et al. unpublished). It was therefore a surprise to find putative meiotic dividing forms and gametes among trypanosomes recovered from proboscides of tsetse infected with *T. simiae* and here we describe these stages as well as providing a detailed account of the development of *T. simiae* in its tsetse vector.

## Methods

### Trypanosomes and tsetse flies

Procyclic forms of *T*. *simiae* strain TV008 (GMOS/GM/85/SAMIWOLO-008), originally isolated from the midgut of an infected tsetse fly (*Glossina morsitans submorsitans*) in The Gambia in 1985 [19], were grown in modified Cunningham’s medium (CM) [20, 21] at 27 °C. Pupae of *Glossina pallidipes* were kindly supplied by the tsetse-rearing facility of IAEA, Vienna; pupae and emerged flies were kept at 25 °C and 70% relative humidity. New emergents were placed in separate cages according to sex and given an infected blood meal for their first feed 24–48 h post-eclosion; thereafter, they were fed on sterile defibrinated horse blood supplemented with 1 mM dATP [22] via a silicone membrane. The infective blood meal contained approximately 10^7^ procyclic trypanosomes ml^−1^ in CM with an equal volume of washed horse red blood cells resuspended in Hank’s Balanced Salt Solution, supplemented with 10 mM l-glutathione [23] to inactivate reactive oxygen species.

### Fly dissection

Cold-anaesthetized flies were dissected 19 to 31 days after infection. The head of the fly was removed with a scalpel blade and retained for subsequent dissection of the proboscis. The whole alimentary tract, from the proventriculus to the rectum, was dissected in a drop of phosphate-buffered saline (PBS) and viewed as a wet mount under phase contrast (× 100 magnification) to determine whether trypanosomes were present; positive midguts were divided into mid-posterior and anterior sections and pooled separately in 500 µl CM. Proventriculi were removed from infected midguts and pooled in 50 µl CM. Proboscides from flies with a midgut infection were dissected into a separate drop of PBS and teased apart to separate the component parts, carefully removing and discarding the labium. The labrum and hypopharynx were pooled in 50 µl CM. All samples were incubated at room temperature for at least 10 min to allow trypanosomes to escape from tissue; in addition, the anterior and mid-posterior midgut samples were passed through a 100-µm filter to remove tissue and then washed twice in PBS before resuspending in 500 µl PBS. Fifty-microlitre aliquots of each sample were mixed with an equal volume of 4% w/v paraformaldehyde in PBS and incubated at room temperature for 30 min. Volume was increased to 200 µl with PBS and cells placed in a Cytospin (1350 rpm for 5 min), air dried, mounted in Vectashield (Vector Labs) containing 4’,6-diamidino-2-phenylindole (DAPI) and viewed immediately. Cibaria from flies with a midgut infection were dissected into a separate drop of PBS and viewed as a wet mount.

### Transfection

Plasmid constructs previously designed for integration into the *PFR1* or *HOP1* genomic loci of *T. brucei* [13] were used for transfection of *T. simiae*. In brief, the *PFR1* construct introduced a C-terminal *YFP* fusion of *PFR1* (*Tb927.3.4290*), while the *HOP1* construct introduced an N-terminal *YFP* fusion of *HOP1* (*Tb10.70.1530*). Successful integration of these constructs thus relied on sufficient homology between the *PFR1* and *HOP1* genes of *T. simiae* and *T. brucei*. Transgenes were expressed via read-through transcription from the endogenous promotors and regulated via the 3′ UTR; while *PFR1* is expressed constitutively, *HOP1* is expressed only during meiosis and therefore the 3′ UTR of *YFP::HOP1* was left unaltered as this region is likely to determine developmentally regulated expression. The antibiotic resistance genes used for selection of transfectants were expressed constitutively. Procyclic trypanosomes were transfected by electroporation (Amaxa program X-01) with 10–20 µg of the linearised plasmids and were selected with either hygromycin (25 µg/ml) or puromycin (0.25 µg/ml). The *PFR1::YFP* transfected line had a fluorescent flagellum and was successfully fly transmitted. Procyclics of the *YFP::HOP1* line were non-fluorescent as expected but failed to develop beyond the midgut during fly transmission and the line died out in culture.

### In vitro development of proventricular trypanosomes

Flies were dissected 27 days after infection with either wild-type *T. simiae* TV008 or a *PFR1::YFP* transfected line. The proventriculus was separated from the foregut and midgut using forceps and hypodermic needles and placed in a tube containing CM with 1 × anti-contamination cocktail (ACC) [24]. Aliquots of this trypanosome suspension were then added to 1-ml wells of CM with 1 × ACC, each well containing a sterile 10–13-mm-diameter round glass coverslip. Plates were incubated at 27 °C and coverslips removed at time intervals and processed as previously described [25].

### Immunofluorescence

Fly proboscides were dissected directly into CM as above and the supernatant fixed for 30 min in 4% w/v paraformaldehyde in PBS at room temperature. The immunofluorescence protocol of [15] was followed to identify the basal body using YL1/2 antibody which recognises tyrosinated α-tubulin [26].

### Imaging and measurements

**Fig. 1:**
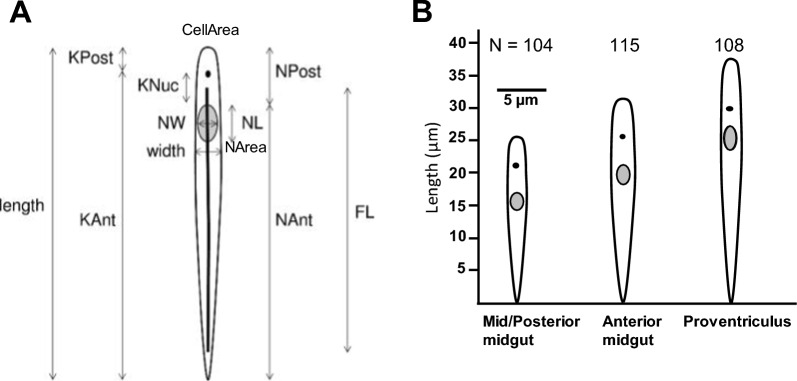
Trypomastigote morphology. **A** The dimensions measured are as indicated: length = total cell length; width = maximum cell width; KNuc = distance between the kinetoplast and nucleus; KPost, NPost = distances from the cell posterior to the kinetoplast or nucleus respectively; KAnt and NAnt, the distances from the cell anterior to the kinetoplast or nucleus, respectively, were derived by subtracting KPost or NPost from the total cell length; NL, NW, NArea = nucleus length, width and area respectively; CellArea = area of cell body. In addition, the length of the paraflagellar rod of the flagellum (FL) was measured in the *PFR1::YFP* transfected line of *T. simiae*. CellArea and NArea were determined by drawing around the cell body or nucleus, respectively. **B** Schematic of changes in trypomastigote morphology in midgut and proventricular trypanosomes. Dimensions of each cell (total = 327) based on mean measurements (µm) for N trypanosomes (Table 1); y axis length, scale bar width

Stained slides were viewed using a DMRB microscope (Leica) equipped with a Retiga Exi camera and Volocity version 4.1 software (Improvision). Each image was photographed under phase contrast and UV fluorescence at 400 × magnification. Digital images were collated and mensural data were collected and analysed using ImageJ software (Version 1.41) (http://rsb.info.nih.gov/ij/); measurements were as previously described [18, 25], with the addition of cell and nuclear area, as shown in Fig. 1A. Data are compiled in Additional file 3: Tables S1 and S3.

### Statistical analysis

Mensural data (11 variables, see Fig. 1A and Additional file 3: Table S3) for 1044 trypanosomes with a single nucleus (excluding cells with two or more nuclei but including cells with one or two kinetoplasts) from midgut, proventriculus and proboscis were subjected to principal components analysis (PCA) using the statistical package IBM SPSS Statistics for Windows, version 26. Three uncorrelated factors, PC1, PC2 and PC3, accounted for 78% of the variance (PC1 36%, PC2 27%, PC3 15%; Additional file 3: Table S2). Extracted scores for these three factors were plotted for each trypanosome (PC1 vs PC2, PC1 vs PC3). A subset consisting of 327 midgut and proventricular cells was subjected to a multivariate analysis using Bonferroni correction to reduce the chances of obtaining false-positive results for the comparison between tsetse tissue of the measurements of the 11 dimensions of cells.

## Results

### Tsetse infection with *Trypanosoma simiae*

Tsetse infections were initiated with cultured *T. simiae* procyclics rather than bloodstream forms to avoid the use of experimental animals. Flies were dissected from 19 days after infection, allowing time for completion of development to infective metacyclics, which typically takes a minimum of 2 weeks. Of 45 *Glossina pallidipes* fed an infected blood meal containing wild-type *T. simiae* and dissected on days 19–28 post infection, 42 (93%) had trypanosomes in the midgut and in 14 of these flies, and trypanosomes were also present in the region of the cibarium (14/42, 33%), though only three had trypanosomes within the cibarium itself. Twenty-three flies with an infected midgut also had an infected proboscis (transmission index 23/42 = 55%). A variety of trypanosome cell types were seen in and around the labrum of the proboscis, both attached and unattached (Additional file 1: Movie S1), but trypanosomes did not usually form the discrete rosettes observed for *T. congolense* [18]. Trypanosomes were also found in the hypopharynx (3 of 7 proboscides examined; Additional file 2: Movie S2), but it was difficult to ascertain cell type in the live preparations and whether the trypanosomes were free or attached.

### Trypanosome cell types in the midgut and proventriculus

To further investigate the cell types present in different fly organs, trypanosomes from the midgut (anterior and mid/posterior sections), proventriculus and proboscis were fixed and stained with DAPI to visualize the nucleus and kinetoplast. In addition, cell structures containing tyrosinated tubulin were visualized by immunofluorescence using the YL1/2 antibody, which serves as a marker for the basal body and newly formed microtubules [26–29]. As well as wild-type trypanosomes, a genetically modified line of *T. simiae* TV008 carrying a *PFR1::YFP* fusion allowed the paraflagellar rod (PFR) inside the flagellum to be visualised by fluorescence microscopy.

The majority of trypanosomes in the midgut and proventriculus were of trypomastigote morphology, where the kinetoplast is posterior to the nucleus, while trypanosomes from the proboscis were much more variable, with epimastigotes (kinetoplast anterior to the nucleus) also present. Measurements of 11 dimensions for a total of 327 midgut and proventricular cells were made (Fig. 1A; Table 1) and subjected to multivariate analysis with Bonferroni correction. Surprisingly, the majority of measurements were significantly different between trypanosomes from the mid/posterior versus anterior midgut (9 of 11 dimensions) and anterior midgut versus proventriculus (8 of 11 dimensions); mid/posterior versus proventricular cells were significantly different for 10 of the 11 dimensions (Table 1). Thus trypanosomes from the mid/posterior midgut, anterior midgut and proventriculus were significantly different in morphology (Fig. 1B), as described in more detail in the next paragraph.

**Table 1:** Morphometry of *Trypanosoma simiae* trypomastigotes from the midgut and proventriculus

Measurement	Mid/posterior midgut	Anterior midgut	Proventriculus
Length	25.41 ± 0.39a	31.37 ± 0.46b	37.48 ± 0.31c
Width	1.91 ± 0.04a	2.19 ± 0.04b	1.87 ± 0.03a
KPost	4.19 ± 0.18a	5.64 ± 0.19b	7.19 ± 0.20c
KNuc	3.52 ± 0.14a	3.81 ± 0.14a	2.31 ± 0.08b
NPost	8.59 ± 0.23a	10.30 ± 0.21b	10.22 ± 0.24b
NL	2.75 ± 0.05a	3.06 ± 0.05b	3.66 ± 0.05c
NW	1.25 ± 0.02a	1.23 ± 0.05a	1.09 ± 0.02b
KAnt	21.21 ± 0.30a	25.73 ± 0.38b	30.30 ± 0.21c
NAnt	16.81 ± 0.31a*	21.07 ± 0.44b*	27.26 ± 0.22c
CellArea	33.18 ± 0.65a	43.56 ± 0.74b	43.89 ± 0.59b
NArea	3.16 ± 0.06a	3.44 ± 0.07b	3.63 ± 0.07b
N	104	115	108

Measurements as in Fig. 1A. Mean ± SE in µm. Within rows, means with different letters are significantly different (*P* < 0.001), except for * (*P* = 0.011)

There was a progressive increase in the length of the cell body and nucleus (Length, NL; *P* < 0.001) among trypanosomes from the mid/posterior to anterior midgut through to the proventriculus (Fig. 1B; Table 1); the distance between the kinetoplast and cell posterior (KPost) and cell anterior (KAnt) also progressively increased. The distance between the nucleus and kinetoplast (KNuc) and nuclear width (NW) were similar between cells from the mid/posterior and anterior midgut but were significantly reduced (*P* < 0.001) in proventricular cells as the nucleus became elongated and sausage-shaped. Cell and nuclear areas, as well as the distance of the nucleus to the posterior of the cell (NPost), were significantly larger (*P* < 0.001) in anterior midgut and proventricular trypanosomes compared to mid/posterior midgut trypanosomes. The proventricular cell population was uniform and consisted of long trypomastigotes with an elongated nucleus. No dividing cells were found in this population whereas occasional dividing cells were encountered in the midgut population (6 dividing trypanosomes in mid/posterior midgut and 7 in the anterior midgut).

### Trypanosome cell types in the proboscis

**Fig. 2:**
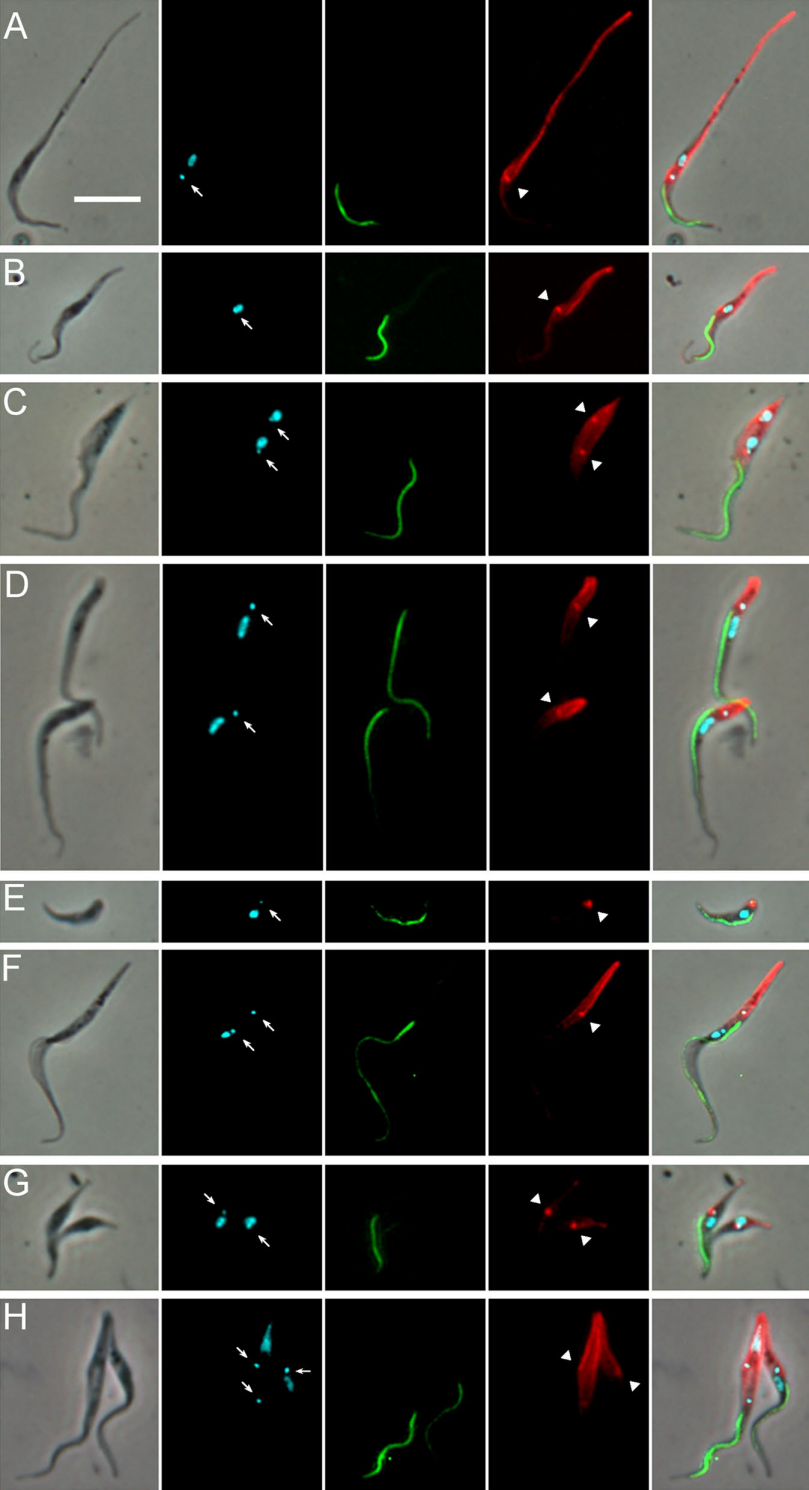
Images of trypanosomes (*Trypanosoma simiae* TV 008 *PFR1::YFP*) from proboscis. From L to R: phase contrast, DAPI false coloured cyan for clarity, YFP, TRITC immunofluorescence with YL1/2 antibody, merge. Arrows: kinetoplasts; arrowheads, basal bodies. **A** Epimastigote with an extremely long posterior. **B** Epimastigote with a short posterior. **C** 2K2N dividing epimastigote. **D** Two trypomastigotes. **E** Metacyclic. **F** 2K1N dividing trypomastigote. **G** 2K2N dividing trypomastigote-epimastigote. **H** 3K2N dividing epimastigote-epimastigote-trypomastigote. Scale bar = 10 µm

Trypanosomes were extracted from pooled tsetse proboscides from flies dissected on days 27, 28 and 31 post infection and stained preparations examined by microscopy to identify the cell types present (Table 2). Among 1463 proboscis trypanosomes examined, the 1K1N epimastigote was the most numerous cell configuration, comprising over 40% (602 of 1463 trypanosomes); such cells had the kinetoplast anterior or juxtaposed to the nucleus and were classified into two types based on the length of the posterior, which was extremely elongated in about one third of cells (204 of 602, 34%; Table 2; Fig. 2A). The posterior of these cells stained intensely with the YL1/2 antibody (Fig. 2A, B), indicating large quantities of tyrosinated tubulin present in the subpellicular microtubules that form the supporting scaffold, and therefore recent growth of the cell posterior. Large numbers of dividing epimastigotes were also evident, having two kinetoplasts and sometimes also two nuclei in the configuration NKNK from the posterior of the cell (90 of 1463, 6.2%) (Table 2; Fig. 2C).

**Table 2:** *Trypanosoma simiae* cell types from the tsetse proboscis

Cell type	No.	%
Epimastigote 1K1N	398	27.2
Epimastigote with extremely elongated posterior 1K1N	204	13.9
Dividing epimastigote 2K1N or 2K2N	90	6.2
Trypomastigote 1K1N	283	19.3
Metacyclic 1K1N	8	0.5
Dividing trypomastigote 2K1N or 2K2N (conformation NKNK)	7	0.5
Epimastigote to trypomastigote 2K1N or 2K2N (conformation NKKN)	29	2
Trypomastigote to epimastigote 2K1N or 2K2N (conformation KNNK)	3	0.2
Zoid 1K0N	19	1.3
Putative sexual stages		
Meiotic divider 2K1N	162	11.1
Meiotic divider 2K2N, 3K2N or 4K2N	23	1.6
Meiotic intermediate 3N (unequal sizes) with variable no. of K	10	0.7
Meiotic intermediate 2N (unequal sizes) with variable no. of K	5	0.3
1-5K2N	47	3.2
3-4K1N	15	1
Gamete 1K1N	100	6.8
Gamete 2K1N	6	0.4
2-3K2N epimastigote to 1-2K1N gamete	7	0.5
3K2N dividing epimastigote to trypomastigote/epimastigote	11	0.8
Unclassified	33	2.3
Total	1463	100

Cell types recovered from tsetse proboscides infected with *T. simiae* TV008 wild type and dissected days 27–31 post infection. *N* nucleus, *K* kinetoplast

A further ~ 20% (291/1463) of proboscis trypanosomes were trypomastigotes, with the kinetoplast posterior to the nucleus (Fig. 2D). Only eight of these were classified as metacyclics, as judged by their morphology (small non-dividing trypanosome with short body, lack of free flagellum and kinetoplast positioned close to the posterior end of the cell; Fig. 2E). As *T. simiae* metacyclics develop in the hypopharynx [1], it is probable that they were poorly represented because the hypopharynx was not disrupted during dissection and was often only lightly infected (Additional file 2: Movie S2). As trypanosomes had ample time for full development to metacyclics in the hypopharynx, a possible reason for light infection is that the *T. simiae* strain used, which originates from The Gambia in West Africa, was not well adapted to the fly species used, *G. pallidipes*, which is of East African origin, but we have limited experience of the development of other strains of *T. simiae*.

**Fig. 3:**
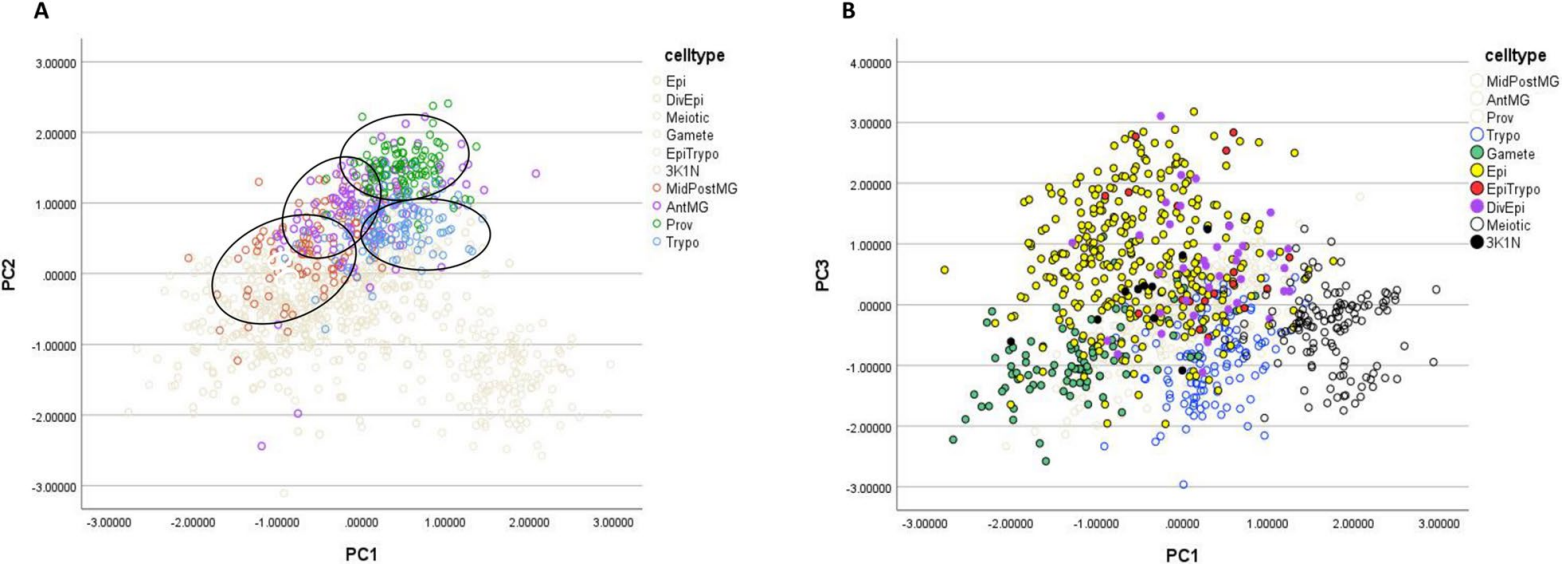
Principal components analysis (PCA): 1044 cells with a single nucleus from all tsetse tissues (midgut, proventriculus and proboscis) were analysed by PCA. **A** Comparison of 443 trypomastigotes from the mid/posterior midgut (104, MidPostMG), anterior midgut (115, AntMG), proventriculus (108, Prov) and proboscis (116, Trypo). For mensural data see Additional file 3: Table S1. **B** Comparison of 717 trypanosomes from the proboscis. Cell types: trypomastigotes (116, Trypo, gametes (93, Gamete), epimastigotes (315, Epi), epimastigote-trypomastigote dividers 2K1N (18, EpiTrypo), epimastigote dividers 2K1N (36, DivEpi), meiotic dividers 2K1N (129, Meiotic) and meiotic intermediates (10, 3K1N). For mensural data see Additional file 3: Tables S1 and S3; for PCA weightings see Additional file 3: Table S2

Trypomastigotes from the proboscis differed from those in the midgut and proventriculus as the distance between the kinetoplast and nucleus (KNuc) in proboscis trypomastigotes (0.99 ± 0.05 µm, *N* = 116) was less than that for trypomastigotes from the midgut and proventriculus (3.67 ± 0.14 µm, *N* = 219; 2.31 ± 0.08 µm, *N* = 108, respectively), and these organelles were closer to the posterior end of the body (KPost: 3.22 ± 0.18 µm; NPost: 4.74 ± 0.17 µm) compared with trypomastigotes from the midgut (KPost: 4.95 ± 0.19 µm; NPost: 9.49 ± 0.22 µm) and proventriculus (KPost: 7.19 ± 0.20 µm; NPost: 10.22 ± 0.24 µm) (Additional file 3: Table S1). To analyse all 11 dimensions simultaneously, we used principal components analysis (PCA), which demonstrated some clustering of trypomastigotes by their tissue location (Fig. 3A). The clusters are not entirely separate and show some overlap, as the four trypomastigote populations are not confined to particular regions, but move from the mid/posterior midgut to the anterior midgut and proventriculus, and thence to the proboscis. Three principal components accounted for 79% of the variance between cells (PC1 37%, PC2 27%, PC3 15%); PC1 was correlated with cell area (CellArea), nucleus area (NArea), nucleus length (NL) and the distance of the nucleus from the cell anterior (NAnt), while PC2 was correlated with KNuc and KAnt and PC3 with KPost and NPost (Additional file 3: Table S2).

Some proboscis trypomastigotes were undergoing cell division as they had two kinetoplasts and sometimes also two nuclei (7 of 1463 cells; Table 2). Some of these were trypomastigote-trypomastigote dividers (Fig. 2F), while others were asymmetric epimastigote-trypomastigote dividers (Fig. 2G, H). In addition, numerous other trypanosomes with multiple kinetoplasts and/or nuclei were observed, some of which resembled sexual stages previously observed in *T. brucei*; these are described in the next section.

**Fig. 4:**
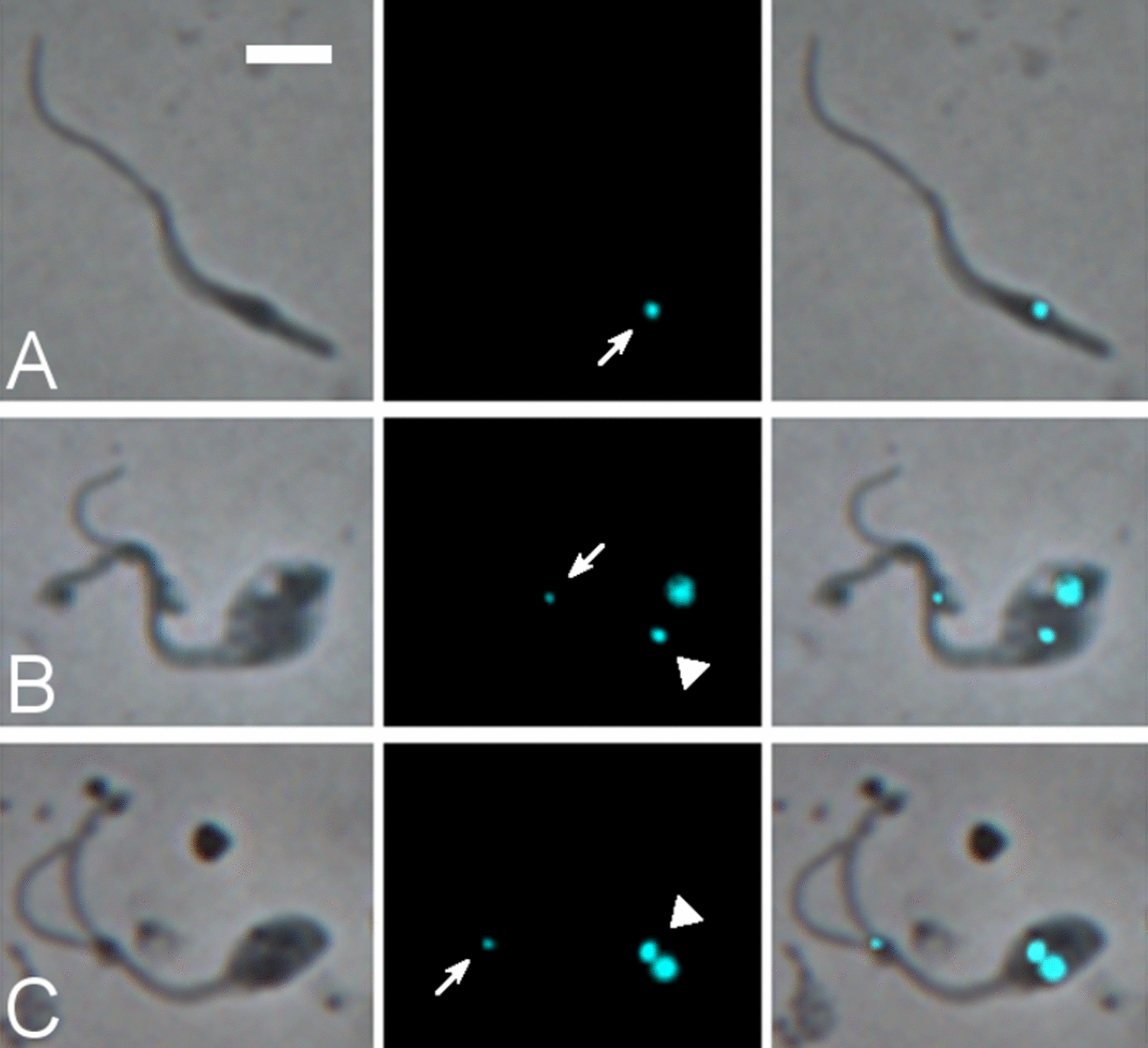
Zoid trypanosomes (*T. simiae* TV008 wild type) from proboscis. From L to R: phase contrast, DAPI false coloured cyan for clarity, merge. Arrows: kinetoplast of zoid; arrowheads, kinetoplast of 1K1N trypanosome. **A** Zoid. **B**, **C** Zoids apparently arising from 1K1N trypanosomes with an extra, extremely anterior kinetoplast. Scale bar = 5 µm

An intriguing observation was the presence of 1K0N trypanosomes (zoids) (Fig. 4A) among trypanosomes in the proboscis at relatively high frequency (1.3%; Table 2). Zoids may have arisen from 2K1N cells with one extremely anterior kinetoplast (Fig. 4B, C). Zoids were not previously noticed among *T. brucei* trypanosomes developing in the salivary glands [30] or *T. congolense* developing in the proboscis [18], and thus this is the first time we have recorded zoids in tsetse infections. In previous studies we have searched through thousands of trypanosome images and applied a threshold of 1% in assuming that any particular cell type occurs frequently enough to be a biologically relevant stage. The biological significance of zoids in *T. simiae* is unknown.

### Identification of putative sexual stages of *T. simiae*

**Fig. 5:**
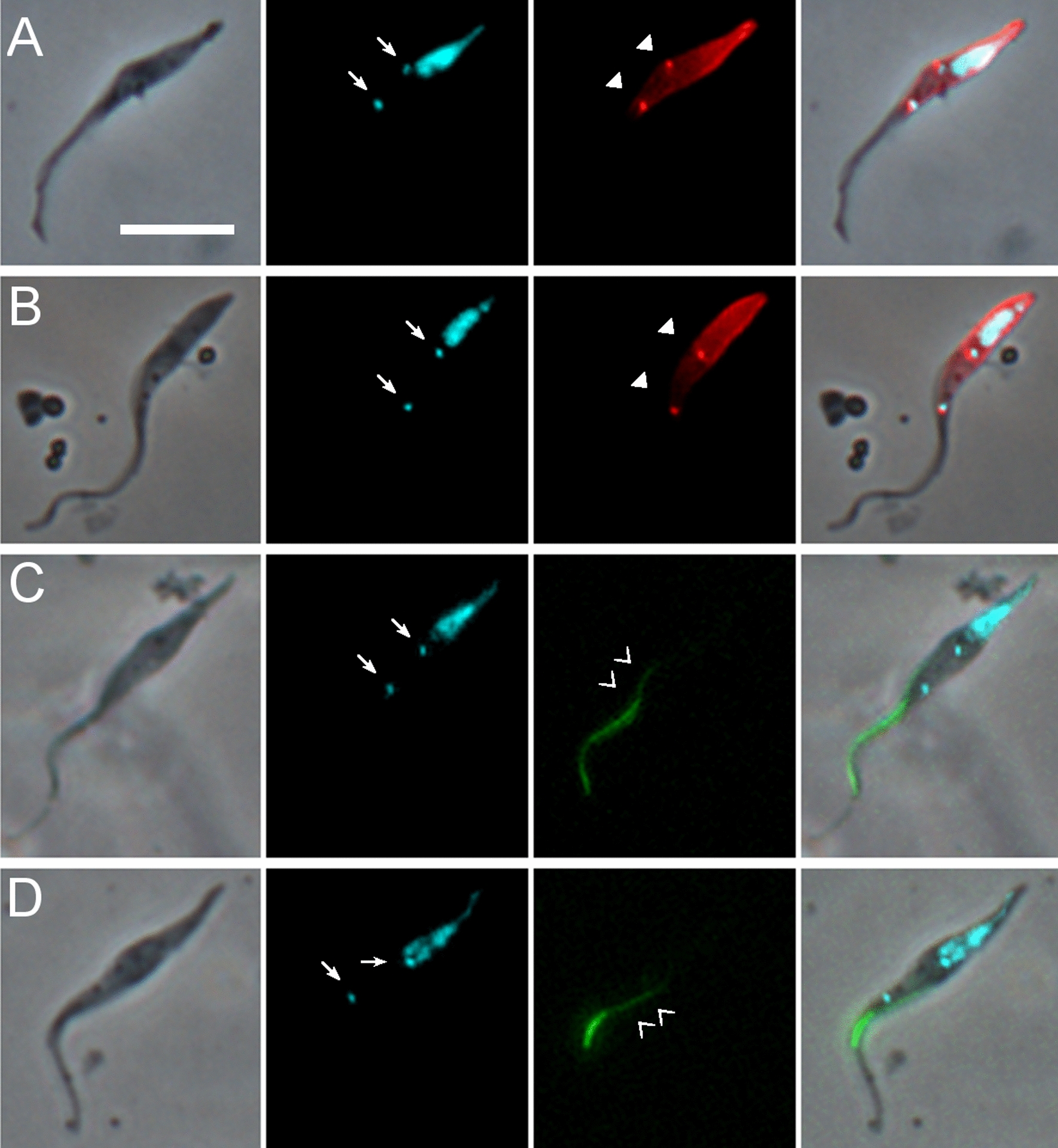
Images of putative meiotic dividers. **A**, **B** Wild-type *T. simiae*. From L to R: Phase contrast, DAPI false coloured cyan for clarity, TRITC immunofluorescence with YL1/2 antibody, merge. Arrows: kinetoplasts; arrowheads: basal bodies. **C**, **D** *PFR1::YFP* line of *T. simiae*. From L to R: phase contrast, DAPI false coloured cyan for clarity, YFP, merge. The PFR of the posterior (daughter) flagellum (double chevrons) was usually less fluorescent than that of the anterior flagellum. Scale bar = 10 µm

**Fig. 6:**
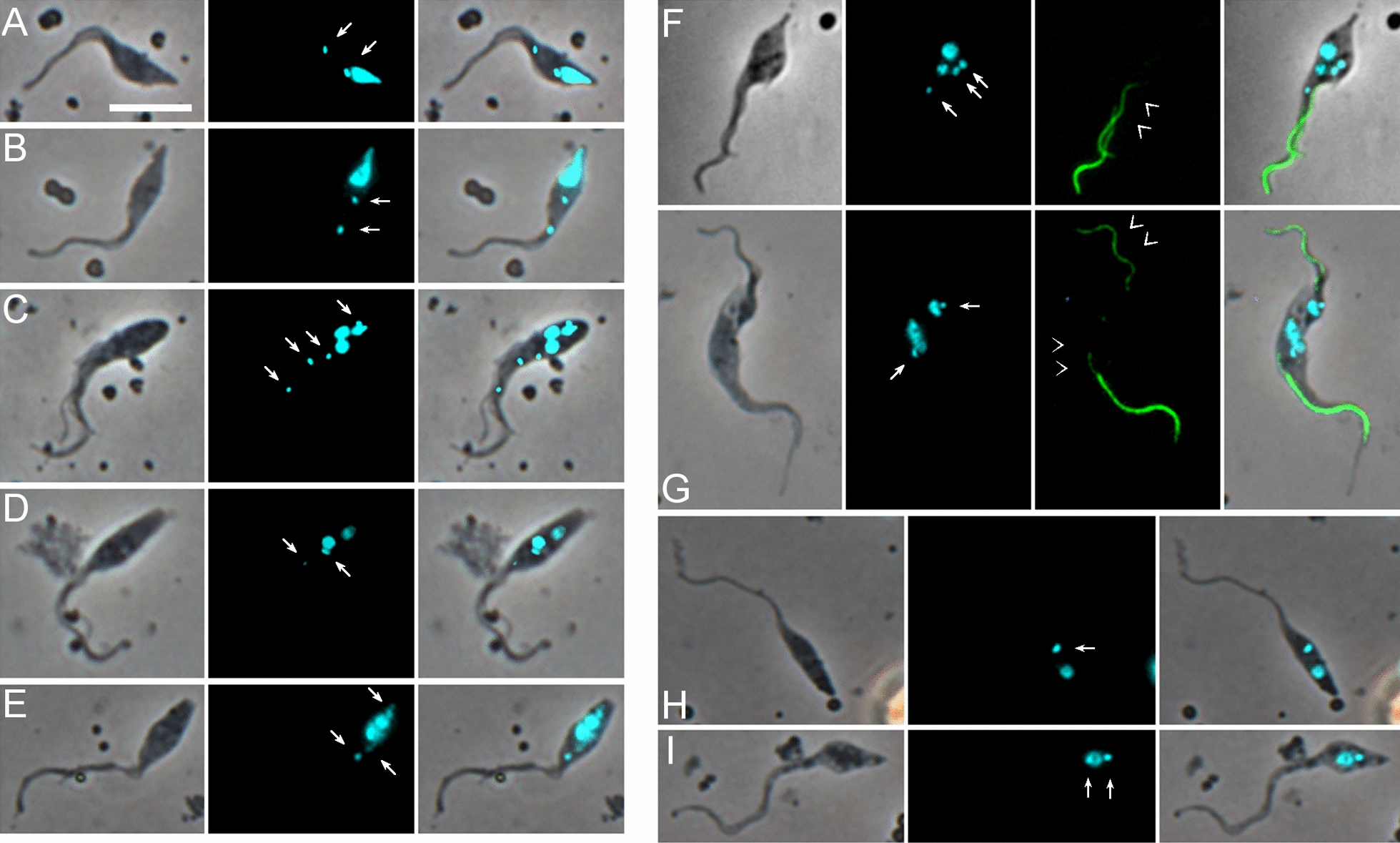
Putative meiotic intermediates and gametes. **A–E** Wild-type *T. simiae* stained with DAPI. From L to R: phase contrast, DAPI false coloured cyan for clarity, merge. Arrows: kinetoplasts. **A**, **B** Putative meiotic dividers with large posterior nucleus. **C** Putative meiotic intermediate with three nuclei and four kinetoplasts. **D**, **E** Putative meiotic intermediates with two nuclei of different fluorescence intensities, i.e. of different sizes; in **D** the posterior kinetoplast is more intensely fluorescent than the anterior and elongated, suggesting that it may be about to divide; in **E** it is uncertain whether the most posterior fluorescent spot is truly a kinetoplast. **F**, **G** *PFR1::YFP* line of *T. simiae*. Putative meiotic intermediates producing daughter cells, which may be gametes. From L to R: phase contrast, DAPI false coloured cyan for clarity, YFP, merge. Double chevrons indicate the PFR of the daughter flagellum, which usually has lower intensity fluorescence compared to the original flagellum. In **F** the identity of the nucleus and kinetoplast(s) of the daughter cell is uncertain, though a new flagellum is evident, judging from the length of visible PFR; in **G** the original cell is already replicating again, judging by the elongation of the nucleus and presence of a new flagellum, though only a single kinetoplast is visible. **H**, **I** Putative 1K1N and 2K1N gametes, respectively. Wild-type *T. simiae* stained with DAPI. From L to R: phase contrast, DAPI false coloured cyan for clarity, merge. Arrows: kinetoplasts. Scale bar = 10 µm

A prominent cell type, comprising 11.1% (162/1463) of proboscis trypanosomes, resembled the meiotic dividing cells previously identified in *T. brucei* [13], having a large nucleus that was positioned close to, and usually filling, the posterior end of the cell, and two kinetoplasts (Figs. 5 and 6A, B). Both kinetoplasts were adjacent to basal bodies stained by the YL1/2 antibody, which recognises tyrosinated α-tubulin (Fig. 5A, B). In addition, the presence of two flagella was verified in the genetically modified line *T. simiae* TV008 *PFR1::YFP* (Fig. 5C, D); the PFR of the posterior (daughter) flagellum was usually less fluorescent than that of the anterior flagellum, as also observed for *T. brucei* [13]. *Trypanosoma brucei* cells with such morphology were shown to express three different meiosis-specific proteins in the nucleus [13], verifying their identity as meiotic dividers, but here our attempt to fly transmit a genetically modified line of *T. simiae* carrying a fusion of *YFP* with the meiosis-specific gene *HOP1* was unsuccessful, as the trypanosome clone failed to develop beyond the midgut during fly transmission. Thus, the identification of these cells as meiotic dividers currently rests on morphology alone.

The putative meiotic dividers formed a separate cluster from other 2K1N epimastigotes in PCA (Fig. 3B). These cell types differed in terms of the area and position of the nucleus: mean NArea meiotic dividers 7.31 ± 0.13 µm^2^ compared to 3.23 ± 0.20 µm^2^ in 2K1N epimastigotes; mean NPost meiotic dividers 0.84 ± 0.11 µm compared to 6.20 ± 0.91 µm in 2K1N epimastigotes (Additional file 3: Table S3).

In trypanosomes as cell division progresses, the distance between the two kinetoplasts K1 and K2 increases [28], and therefore K1-K2 distance can be used to monitor the progress of meiotic division and investigate associated morphological changes. Correlation analysis showed that as the K1-K2 distance increased, there was a significant lengthening of the cell, yet no significant increase in width or cell area; at the same time the nucleus became longer and slightly narrower without a noticeable change in area (Additional file 4: Fig. S1).

In our previous analysis of meiotic division in *T. brucei* [15], we assumed that the 4C (4 × haploid DNA content) posterior nucleus of the meiotic dividing cell went on to divide into two 2C (2 × haploid DNA content) nuclei, but as the morphology of this cell would resemble that of a dividing epimastigote, it would be difficult to identify unequivocally. We inferred that subsequent division of these nuclei was asynchronous, because trypanosomes containing either one 2C and two 1C nuclei or one 2C and one 1C nucleus were observed; such trypanosomes sometimes had a well-advanced cell cleavage furrow, splitting off a small haploid daughter cell [15]; a further unusual feature of these putative multi-nucleate meiotic intermediates was that they had variable numbers of kinetoplasts. Cells of similar morphology were identified here in *T. simiae* (Table 2; Fig. 6C–G), potentially representing meiotic intermediates. These trypanosomes were observed alongside abundant meiotic dividers in the same microscope fields (Additional file 5: Fig. S2).

We also identified potential gametes among proboscis *T. simiae* trypanosomes by their resemblance to gametes of *T. brucei*, which have a small pear-shaped body and a relatively long flagellum [14, 15]. In *T. simiae* the putative gametes had a small cell body that was wide near the posterior to accommodate the single nucleus and sometimes tapered almost to the anterior end of the cell (Fig. 6H, I); both 1K1N and 2K1N putative gametes were observed, but the majority were 1K1N (100 1K1N and 6 2K1N, Table 2). These putative 1K1N gametes were distinct from other 1K1N epimastigotes in terms of the area of the cell and nucleus and position of nucleus and kinetoplast: mean CellArea, mean NArea 1K1N gametes 22.22 ± 6.93, 1.58 ± 0.57 µm^2^ (respectively) compared to 33.77 ± 0.50, 2.41 ± 0.05 µm^2^ (respectively) in 1K1N epimastigotes; mean NPost, mean KPost 1K1N gametes 2.59 ± 1.46, 4.53 ± 1.66 µm (respectively) compared to 10.04 ± 0.33, 11.93 ± 0.34 µm (respectively) in 1K1N epimastigotes (Additional file 3: Table S3). The putative gametes formed a distinct cluster in PCA (Fig. 3B).

In summary, the presence of putative meiotic intermediates and gametes suggests that meiosis is part of the *T. simiae* life cycle in the tsetse proboscis, just as it is part of the *T. brucei* life cycle in the tsetse salivary glands [13, 14].

### In vitro development of proventricular trypanosomes

**Fig. 7:**
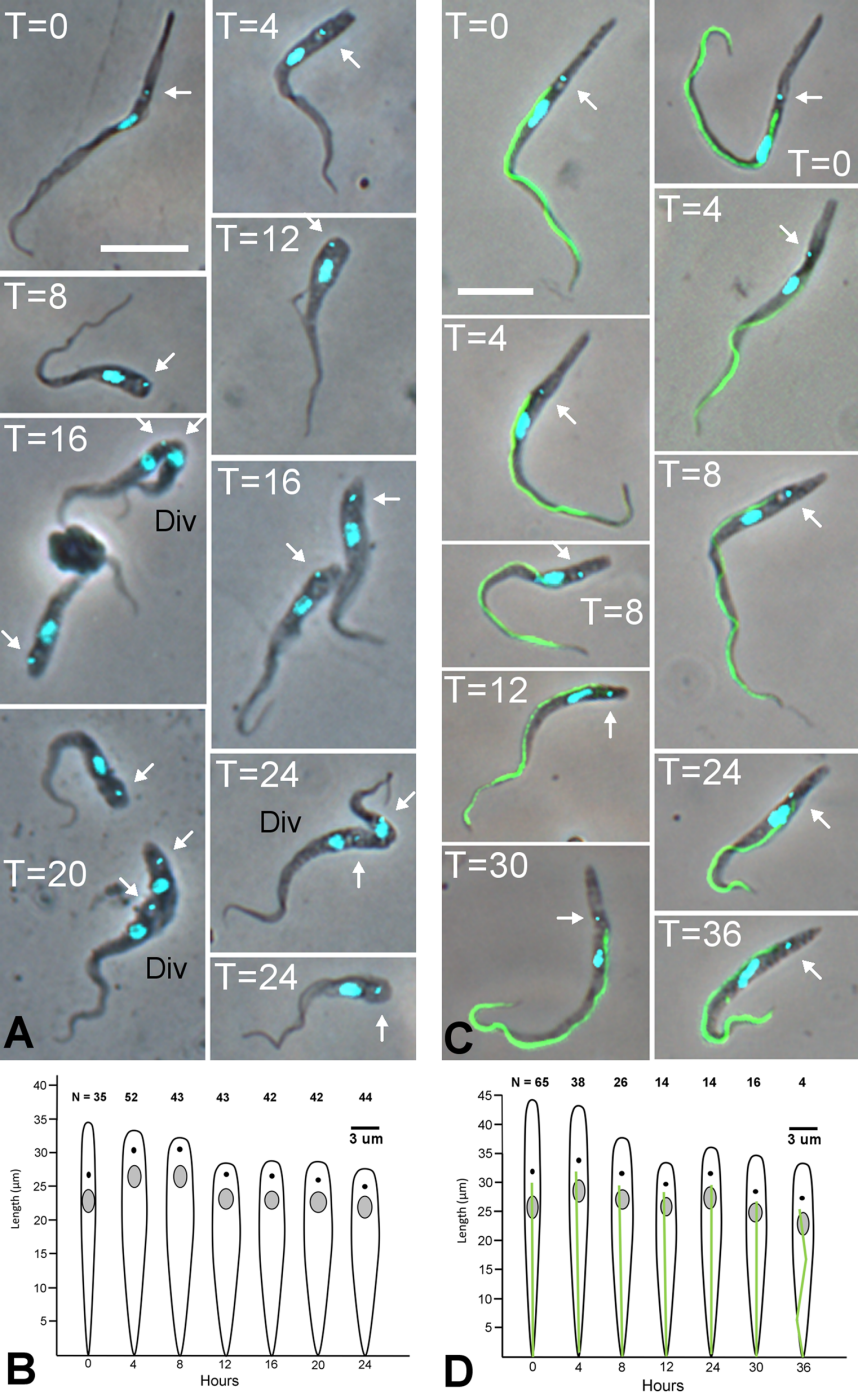
In vitro development of *T. simiae* proventricular trypanosomes. **A** Representative images of *T. simiae* cells from a 24-h time course of development of wild-type proventricular trypanosomes in vitro. At T = 0 h proventricular trypanosomes were put into wells of culture medium and sampled every 4 h. By *T* = 16 h some cells had almost completed cell division (Div). Kinetoplasts are indicated by arrows. Scale bar = 10 µm. **B** Morphological changes of 1K1N cells recorded over the 24-h time course; drawings based on mean measurements of N cells; y axis length, scale bar width. **C** Representative images of *T. simiae* TV008 *PFR1::YFP* cells from a 36-h time course of development of proventricular trypanosomes in vitro. Each image is a merge of phase contrast, DAPI (false coloured cyan for clarity) and YFP images. Kinetoplasts are indicated by arrows. At *T* = 0 proventricular trypanosomes were put into wells of culture medium and sampled at intervals. The progression of this time course with genetically modified trypanosomes was slightly different to the wild type and no dividing cells were seen, perhaps because trypanosome numbers were smaller. Scale bar = 10 µm. **D** Morphological changes of 1K1N cells recorded over the 36-h time course; drawings based on mean measurements of N cells. Dimensions of each cell (total = 327) based on mean measurements (µm) for N trypanosomes (Table 1); y axis length, scale bar width. For mensural data see Additional file 3: Table S4

As it would be difficult to identify newly arrived, migratory trypanosomes in the proboscis of a live tsetse fly, we studied the development of proventricular trypanosomes in vitro, an approach successfully applied to *T. congolense* previously [25]. Dissected proventriculi were pooled to release proventricular trypanosomes, which were then incubated overnight in wells of culture medium containing a sterile glass coverslip. The long proventricular trypomastigotes attached to the coverslip and gradually became shorter and stouter, as previously observed for *T. congolense* [25] (Fig. 7A). However, attachment was not as rapid as for *T. congolense*, where the cells attached almost as soon as they touched the coverslip; for *T. simiae*, trypanosomes moved around and appeared to “probe” with the tip of their flagellum for 30 – 60 min before finally starting to attach. After 8 h, some cells initiated cell division, again as previously observed for *T. congolense* [25], but between 24 and 48 h increasing numbers of cells became misshapen and bloated and cultures died out by 72 h.

Image and morphometric data were collected from an overlapping sequential time course from *T* = 0 to *T* = 24 h. At *T* = 0 proventricular trypanosomes formed a uniform population of long trypomastigotes with a single nucleus and kinetoplast (1K1N). At *T* = 8 h 2K1N cells started to appear and by T = 16 h some cells had already completed cell division, producing a smaller daughter cell (Fig. 7A); approximately equal numbers of epimastigote and trypomastigote morphologies were observed among the daughter cells. Figure 7B shows the morphological changes in 1K1N trypanosomes from T = 0 to *T* = 24 h. Over the first 4 h, the distance of the cell posterior from the nucleus and kinetoplast decreased, while the distance between these organelles and the cell anterior increased; the cells became noticeably wider. From 8 to 12 h, the cells shortened, with the reduction in length reflected in decreased distance between the nucleus and cell anterior; the nucleus, at first elongated, became rounded. The morphology of the cells remained relatively constant from 12 to 24 h (Fig. 7B).

Previous analysis of a similar time course of development of *T. congolense* proventricular trypanosomes showed that the paraflagellar rod (PFR) of the flagellum shortened by about 30%, accompanied by a noticeable accumulation of the PFR protein, PFR1, visualised by tagging PFR1 with YFP [24]. The same approach was used here to tag PFR1 of *T. simiae* TV008 and follow remodelling of the flagellum during development of proventricular forms in vitro (Fig. 7C, D). For unknown reasons, the progression of this time course with genetically modified trypanosomes was slightly different to wild type; the initial proventricular trypanosomes were substantially longer, ~ 45 µm compared to 35 µm for wild type, and no dividing cells were seen, perhaps because trypanosome numbers were smaller. As with the wild-type trypanosomes, the genetically modified trypanosomes showed a similar pattern of morphological changes, with cells becoming shorter and stouter over the time course (Fig. 7C, D). In contrast to *T. congolense* [25], the maximum reduction in PFR length was 10%, which occurred within the first 12 h, and no further reduction was observed up to the end of the 36 h time course; the accumulation of PFR1::YFP was not observed at any timepoint, perhaps because flagellar length reduction was less pronounced in *T. simiae* compared to *T. congolense*.

## Discussion

Paradoxically the virulent pig pathogen *Trypanosoma simiae* is widespread and common in sub-Saharan Africa but little studied compared to its relatives *T. brucei* and *T. congolense*. Here we analysed image and mensural data from both wild-type and genetically modified *T. simiae* during its development in the tsetse midgut, proventriculus and proboscis, confirming that the life cycles of *T. simiae* and *T. congolense* are closely similar as expected, since both species were placed in the same subgenus based on their developmental characteristics in the tsetse vector [1]. The surprising finding was the presence of abundant (putative) sexual stages among *T. simiae* trypanosomes in the proboscis. To date, *T. brucei* is the only tsetse-transmitted trypanosome with experimentally proven capability to undergo sexual reproduction, which occurs in the fly salivary glands [31]. By analogy, sexual stages of *T. simiae* or *T. congolense* are predicted to occur in the proboscis, where the corresponding portion of the developmental cycle, involving proliferation of epimastigotes and the generation of infective metacyclics, takes place. However, no obvious sexual stages were observed in a previous detailed investigation of the *T. congolense* life cycle in tsetse [18], though population genetics evidence points to sexuality in *T. congolense* [16] and hybrids have been identified in the tsetse proboscis in laboratory crosses (Peacock et al., unpublished).

Amongst the various trypanosome morphotypes present in the proboscis were cells with a large posterior nucleus and two anterior kinetoplasts, the characteristic morphological features of trypanosomes in Meiosis I in *T. brucei*, as determined by expression of three meiosis-specific proteins in the nucleus [13]. Our attempt to verify the identity of these putative meiotic dividers in *T. simiae* in a similar way by expression of a YFP-tagged version of HOP1 unfortunately failed, so the identification currently rests on morphology alone. However, some supporting evidence is provided by the co-occurrence, alongside the meiotic dividers in the proboscis, of putative gametes and other meiotic intermediates, again identified by morphological similarity to these sexual stages in *T. brucei* [14, 15].

Experimental verification that the *T. simiae* morphotypes observed are truly sexual stages is essential. Previous experiments to elucidate the sexual cycle of *T. brucei* have relied on genetically modified parental lines to facilitate the identification of hybrid progeny [31–33] rather than the needle-in-haystack approach used initially [12]. The demonstration that *T. simiae* can be genetically manipulated using tools designed for *T. brucei* will facilitate similar experiments involving genetically modified lines.

The development of the subgenus *Nannomonas* trypanosomes *T. simiae* and *T. congolense* in tsetse differs profoundly from that of *T. brucei* in several respects. In all three species, procyclics proliferate in the midgut and colonise the proventriculus, but the population of proventricular forms in subgenus *Nannomonas* is uniform and non-dividing [18], whereas in *T. brucei* proventricular forms undergo an asymmetric division into one short and one long epimastigote [30, 34]. Thus, the differentiation to epimastigote forms occurs after migration to the mouthparts in *T. simiae* and *T. congolense*, rather than before and during migration as in *T. brucei*. This difference allows the differentiation of proventricular forms of *T. simiae* and *T. congolense* to be followed in vitro. In our previous investigation of this process in *T. congolense*, we found that almost as soon as the long proventricular forms were put into culture medium, they attached to the substrate and then underwent dramatic shortening before starting an asymmetric division to produce a small daughter cell [25]. A similar pattern of development was observed here for *T. simiae*, though initial attachment took slightly longer and shortening of the cell body and flagellum did not result in formation of a depot of paraflagellar rod protein, thought to result from the rapid deconstruction of the flagellum [25].

The developmental cycles of all three trypanosome species culminate in the production of infective metacyclics that can be inoculated into the next host with saliva as the fly feeds. In *T. brucei* this is achieved by direct colonisation of the salivary glands via the hypopharynx (the tube that carries saliva from the glands to the tip of the proboscis), whereas in *T. simiae* and *T. congolense* the internal lumen of the proboscis is colonised first before invasion of the hypopharynx. Thus, trypanosomes of all three species need to get into the hypopharynx, presumably via its open end, which is surrounded by finger-like projections [35]. The biological function of the finger-like projections is unknown, but could be to guard against passive entry of microorganisms. In contrast, trypanosomes are motile and in *T. brucei* it is the migratory life cycle stages from the proventriculus—asymmetrically dividing epimastigotes, and long and short epimastigotes—that invade the hypopharynx; these stages have been recovered from both the proventriculus and salivary exudate during early colonisation of the salivary glands [30, 36]. For *T. congolense* and *T. simiae*, it is more difficult to identify the invasive life cycle stages, because salivary exudate will contain a heterogeneous mixture of trypanosomes from the hypopharynx and food canal. Trypanosomes within the hypopharynx can be viewed in live dissected material, and here mostly small trypanosomes, possibly metacyclics, were observed (Additional file 2: Movie S2). Determining the identity of the invasive stage awaits the development of appropriate stage-specific markers.

## Conclusions

To date, *T. brucei* is the only tsetse-transmitted trypanosome with experimentally proven capability to undergo sexual reproduction, which occurs in the fly salivary glands. By analogy, sexual stages of *T. simiae* or *T. congolense* are predicted to occur in the proboscis, where the corresponding portion of the developmental cycle takes place. While no such stages have been observed in *T. congolense*, for *T. simiae* putative sexual stages were abundant in the tsetse proboscis. Although our initial attempt to demonstrate expression of a YFP-tagged, meiosis-specific protein was unsuccessful, the future application of transgenic approaches will facilitate the identification of meiotic stages and hybrids in *T. simiae*.

## Supplementary Information

**Additional file 1: Movie S1.** Tsetse fly labrum infected with *Trypanosoma simiae.* A dense group of trypanosomes is attached to the inner wall of the labrum of the tsetse proboscis.

**Additional file 2: Movie S2.** Tsetse fly hypopharynx infected with *Trypanosoma simiae.* In the first part of the movie, the hypopharynx is seen as a thin tube containing motile trypanosomes, lying adjacent to the tip of the labrum in this preparation. Finger-like projections can be seen at the tip of the hypopharynx. The second part of the movie shows a higher magnification and individual trypanosomes can be seen. These appear to be much shorter than those seen in the labrum (S1_Movie) and may be metacyclics.

**Additional file 3: Table S1.** Morphometry of *T. simiae* 1K1N trypomastigotes in tsetse midgut, proventriculus and proboscis. **Table S2.** Loadings for principal components PC1, PC2 and PC3. **Table S3.** Morphometry of *T. simiae* 1N trypanosomes in tsetse proboscis, including putative sexual stages. **Table S4.** Morphometry of *T. simiae* cells from a time course of development of proventricular trypanosomes in vitro.

**Additional file 4: Fig. S1.** Analysis of meiotic dividers. Correlations and diagram based on measurements of 129 2K1N meiotic dividers. **A** Correlations for 11 measured variables. In linear regression graphs, X-axis is K1-K2 distance (µm) and Y-axis is an individual morphometric (µm); above each graph is the *R*^2^ value, with the Pearson correlation coefficient and *P* value below in brackets. **B** Schematic of progression based on the increasing distance between the two kinetoplasts (K1-K2) and the 11 other morphometrics, some of which changed at the same time. K1 is closest to the cell anterior.

**Additional file 5: Fig. S2.** Images of trypanosomes from the proboscis. These images of part microscope fields give a feel for the abundance of meiotic dividers (*M*) and gametes (*G*) among epimastigotes (*E*) and other cell types from the proboscis. Scale bar = 10 um.

## Data Availability

All data generated or analysed during this study are included in this published article and its supplementary information.

## Abbreviations

CM: Cunningham’s medium
DAPI: 4’,6-Diamidino-2-phenylindole
PBS: Phosphate buffered saline
PCA: Principal components analysis
PFR: Paraflagellar rod
YFP: Yellow fluorescent protein
