# Supplementary material for: Development of the livestock pathogen Trypanosoma (Nannomonas) simiae in the tsetse fly with description of putative sexual stages from the proboscis
Source: Parasit Vectors. 2023 Jul 11;16:231. doi: 10.1186/s13071-023-05847-5 (PMC10337175; doi:10.1186/s13071-023-05847-5)
Supplement: Supplementary file 3 — Additional file 3: Table S1. Morphometry of T. simiae 1K1N trypomastigotes in tsetse midgut, proventriculus and proboscis. Table S2. Loadings for principal components PC1, PC2 and PC3. Table S3. Morphometry of T. simiae 1N trypanosomes in tsetse proboscis, including putative sexual stages. Table S4. Morphometry of T. simiae cells from a time course of development of proventricular trypanosomes in vitro. [file 13071_2023_5847_MOESM3_ESM.pdf]

S1 Table. Morphometry of *T. simiae* 1K1N trypomastigotes in tsetse midgut, proventriculus and proboscis.

The mean ± SE in µm is top line in each box with the range below. L = total cell length; W = maximum cell width; KPost, NPost = distances from the cell posterior to the kinetoplast or nucleus respectively; KAnt and NAnt, distances from the cell anterior to the kinetoplast or nucleus respectively, were derived by subtracting KPost or NPost from the total cell length; KNuc = distance between the kinetoplast and nucleus; NL, NW, NArea = nucleus length, width and area respectively; CellArea = area of cell body. Total no. of cells = 443.

Morphotype	N	L	W	KPost	NPost	KAnt	NAnt	KNuc	NL	NW	NArea	CellArea
Mid/posterior midgut trypomastigotes	104	25.41 ± 0.39<br>15.82-34.80	1.91 ± 0.04<br>1.30-3.19	4.19 ± 0.18<br>0.73-8.07	8.59 ± 0.23<br>3.65-15.24	21.21 ± 0.30<br>13.71-29.71	16.81 ± 0.31<br>9.71-26.27	3.52 ± 0.14<br>0.73-7.08	2.75 ± 0.05<br>1.45-4.06	1.25 ± 0.02<br>0.81-1.79	3.16 ± 0.06<br>1.71-4.88	33.18 ± 0.65<br>18.16-49.03
Anterior midgut trypomastigotes	115	31.37 ± 0.46<br>20.04- 48.56	2.19 ± 0.04<br>1.40-4.15	5.64 ± 0.19<br>1.39-14.79	10.30 ± 0.21<br>4.26-18.25	25.73 ± 0.38<br>16.41-36.48	21.07 ± 0.44<br>13.62-33.10	3.81 ± 0.14<br>0.23-6.68	3.06 ± 0.05<br>1.79-5.16	1.23 ± 0.05<br>0.49-2.28	3.44 ± 0.07<br>2.08-5.80	43.56 ± 0.74<br>27.47-79.92
Proventricular trypomastigotes	108	37.48 ± 0.31<br>28.16-47.21	1.87 ± 0.03<br>1.17-2.88	7.19 ± 0.20<br>2.00-14.05	10.22 ± 0.24<br>3.58-15.29	30.30 ± 0.21<br>25.33-36.83	27.26 ± 0.22<br>22.25-33.41	2.31 ± 0.08<br>0.32-4.22	3.66 ± 0.05<br>2.60-5.06	1.09 ± 0.02<br>0.67-1.62	3.63 ± 0.07<br>2.29-6.62	43.89 ± 0.59<br>25.65-59.44
Proboscis trypomastigotes	116	30.44 ± 0.29<br>19.97-40.26	2.57 ± 0.04<br>1.39-3.84	3.22 ± 0.18<br>0.32-12.93	4.74 ± 0.17<br>2.01-13.40	27.22 ± 0.27<br>17.35-34.60	25.70 ± 0.25<br>16.34-32.53	0.99 ± 0.05<br>0.00-2.34	2.98 ± 0.05<br>1.24-4.37	1.27 ± 0.02<br>0.81-1.96	3.47 ± 0.08<br>0.87-6.33	39.52 ± 0.67<br>27.28-59.73

S2 Table. Loadings for principal components PC1, PC2 and PC3.

Variables contributing most to each principal component are in bold.

**Variable**	**PC1**	**PC2**	**PC3**
CellArea	**0.184**	0.132	0.219
NK	-0.079	**0.253**	-0.060
logLength	0.115	0.221	0.297
logWidth	0.148	-0.176	0.082
logNL	**0.214**	0.033	-0.100
logNW	0.163	-0.160	0.005
logNPost	-0.107	0.196	**0.333**
logNArea	**0.224**	-0.013	-0.072
logNAnt	**0.178**	0.115	-0.154
log KPost	0.045	-0.102	**0.531**
logKAnt	0.080	**0.290**	-0.192

S3 Table. Morphometry of *T. simiae* trypanosomes with a single nucleus in tsetse proboscis, including putative sexual stages.

The mean ± SE in µm is top line in each box with the range below. L = total cell length; W = maximum cell width; K1Post, K2Post, NPost = respectively the distances from the cell posterior to kinetoplast 1 or 2 (if present), or nucleus; K1Ant, K2Ant, NAnt, respectively the distances from the cell anterior to kinetoplast 1 or 2 (if present), or nucleus, were derived by subtracting K1Post, K2Post, NPost from the total cell length; K1Nuc, K2Nuc = distance between kinetoplast 1 or 2 (if present) and the nucleus; K1K2 = distance between kinetoplasts K1 and K2; NL, NW, NArea = nucleus length, width and area respectively; CellArea = area of cell body.

Morphotype	N	L	W	K1Post	K2Post	NPost	K1Ant	K2Ant	NAnt	K1Nuc	K2Nuc
Epimastigote 1K1N	315	28.64 ± 0.33<br>14.23-48.42	2.45 ± 0.03<br>1.15-4.22	11.93 ± 0.34<br>1.85-31.27	-	10.04 ±0.33<br>0.97-28.46	16.71 ± 0.24<br>7.53-32.97	-	18.61 ± 0.23<br>8.50-33.80	-0.24 ± 0.02<br>-1.62-0.00	-
Dividing epimastigote 2K1N	36	28.00±0.88<br>15.52-40.77	3.26±0.08<br>2.18-4.22	11.40±0.85<br>4.42-28.44	8.39±0.87<br>3.20-26.46	6.20±0.91<br>0.59-24.97	15.60±0.68<br>9.44-26.42	19.60±0.69<br>12.32-28.38	21.80±0.73<br>14.27-29.08	2.83±0.57<br>0.00-16.36	0.64±0.19<br>0.51-6.10
Epimastigote-Trypomastigote 2K1N	18	28.69 ± 1.27<br>18.41-38.85	2.78 ± 0.09<br>1.95-3.41	12.52 ± 1.72<br>5.77-26.21	7.98 ± 1.68<br>1.27-21.90	8.61 ± 1.70<br>1.95-22.37	16.17 ± 1.17<br>6.56-23.63	20.71 ± 1.13<br>10.89-27.45	20.08 ± 1.21<br>10.15-27.55	-1.16 ±0.23 -<br>3.95-0.00	0.51 ± 0.11<br>-0.36-1.54
Meiotic divider 2K1N	129	26.71±0.25<br>21.03-33.88	3.50±0.04<br>2.34-4.57	10.49±0.18<br>5.01-15.76	6.72±0.13<br>3.75-11.63	0.84±0.11<br>0.00-8.75	16.22±0.22<br>10.36-21.86	19.99±0.22<br>13.73-25.91	25.87±0.25<br>18.85-33.23	4.15±0.13<br>0.19-4.15	0.77±.08<br>0.65-0.76
Meiotic intermediate 3K1N	13?										
Gametes total 1K1N and 2K1N	93	23.32 ± 3.75<br>13.50-31.59	2.00 ± 0.63<br>1.09-4.73	4.78 ± 2.30<br>0.73-16.01	-	2.60 ± 1.49<br>0.36-10.40	18.53 ± 3.42<br>6.45-27.43	-	20.72 ± 3.27<br>12.47-29.31	-0.70 ± 1.94<br>-13.02-0.00	-
Gamete 1K1N	81	23.16 ± 3.52<br>13.50-31.01	1.89 ± 0.48<br>1.09-3.29	4.53 ± 1.66<br>0.73-12.19	-	2.59 ± 1.46<br>0.69-10.40	18.63 ± 3.10<br>10.83-27.43	-	20.57 ± 3.10<br>12.47-29.31	-0.38 ± 0.64<br>-3.35-0.00	-
Gamete 2K1N	12	24.37 ± 5.09<br>16.86-31.59	2.73 ± 0.98<br>1.60-4.73	6.52 ± 4.53<br>1.82-16.01	2.47 ± 1.60<br>0.46-5.08	2.67 ± 1.69<br>0.36-5.00	17.86 ± 5.27<br>6.45-26.01	21.91 ± 4.29<br>16.04-29.96	21.71 ± 4.26<br>16.20-28.63	-2.91 ± 4.74<br>-13.02-0.00	0.00 ± 1.03<br>-2.68-1.63

S3 table contd.

Morphotype	N	K1K2	NL	NW	NArea	CellArea
Epimastigote 1K1N	315	-	2.21 ± 0.03 0.99-4.62	1.24 ± 0.03 0.69-8.94	2.41 ± 0.05 1.00-7.75	33.77 ± 0.50 13.83-64.45
Dividing epimastigote 2K1N	36	2.42±0.40 0.00-10.54	2.53±0.12 1.15-3.80	1.46±0.05 0.97-2.08	3.23±0.20 1.11-5.90	38.56±1.33 22.59-57.38
Epimastigote-Trypomastigote 2K1N	18	3.91 ±0.24 2.93-6.65	2.72 ± 0.13 1.85-3.60	1.55 ± 0.05 1.15-1.98	3.39 ± 0.18 2.35-5.11	39.77 ± 1.65 28.68-56.51
Meiotic divider 2K1N	129	3.32±0.08 1.15-5.41	5.28±0.10 2.62-8.50	2.10±0.03 1.15-3.09	7.31±0.13 3.56-11.94	40.62±0.53 28.23-58.94
Meiotic intermediate 3K1N						
Gametes total 1K1N and 2K1N	93	-	1.79 ± 0.55 0.87-3.25	0.99 ± 0.25 0.51-1.76	1.59 ± 0.61 0.66-3.66	22.93 ± 7.74 10.65-47.08
Gamete 1K1N	81	-	1.81 ± 0.51 0.87-3.25	0.96 ± 0.22 0.51-1.50	1.58 ± 0.57 0.71-3.66	22.22 ± 6.93 11.26-45.31
Gamete 2K1N	12	3.19 ± 3.41 0.59-11.01	1.60 ± 0.76 0.87-3.19	1.16 ± 0.33 0.65-1.76	1.60 ± 0.84 0.66-3.27	27.73 ± 11.06 10.65-47.08

S4 Table. Morphometry of *T. simiae* cells from a time course of development of proventricular trypanosomes *in vitro*.

At Time = 0 hours, *T. simiae* TV008 proventricular trypanosomes were put into wells of culture medium and sampled every 4 hours. The mean ± SE in µm is top line in each box with the range below. L = total cell length; W = maximum cell width; KPost, NPost = respectively the distances from the cell posterior to kinetoplast, or nucleus; KAnt, NAnt, respectively the distances from the cell anterior to kinetoplast or nucleus, were derived by subtracting K1Post, NPost from the total cell length; KNuc = distance between kinetoplast and the nucleus; NL, NW = nucleus length and width respectively. For *T. simiae* TV008 *PFR1::YFP*, the visible fluorescent paraflagellar rod (PFR) of the flagellum allowed three further measurements: FL, FPost, FAnt = respectively PFR length, distances between cell posterior and PFR posterior end, and distances between cell anterior and PFR anterior end.

Wildtype *T. simiae* TV008

Time		N	L	W	KPost	NPost	KAnt	NAnt	KNuc	NL	NW
0		35	34.43 ± 0.66<br>27.26-40.44	1.52 ± 0.04<br>1.15-1.95	7.35 ± 0.31<br>2.40-11.47	10.37 ± 0.34<br>6.62-14.38	27.08 ± 0.51<br>20.59-31.54	24.07 ± 0.45<br>18.30-28.06	2.19 ± 0.11<br>0.67-3.87	3.24 ± 0.08<br>2.54-4.47	1.18 ± 0.04<br>0.73-1.66
4		52	33.38 ± 0.59<br>25.49-42.39	2.67 ± 0.08<br>1.50-4.23	2.56 ± 0.23<br>0.51-6.71	5.18 ± 0.27<br>2.21-10.34	30.83 ± 0.53<br>23.45-40.08	28.20 ± 0.52<br>20.66-36.46	2.01 ± 0.11<br>0.67-3.87	3.00 ± 0.07<br>2.08-4.57	1.47 ± 0.04<br>0.95-2.14
8		43	32.21 ± 0.61<br>23.07-40.21	3.04 ± 0.07<br>2.24-4.11	1.34 ± 0.12<br>0.16-3.63	4.05 ± 0.12<br>2.76-5.84	30.88 ± 0.58<br>22.70-38.18	28.16 ± 0.56<br>20.31-35.95	2.04 ± 0.08<br>0.87-3.31	3.00 ± 0.07<br>1.95-4.12	1.48 ± 0.05<br>0.99-2.26
12		43	28.52 ± 0.64<br>19.44-35.87	2.96 ± 0.08<br>1.75-4.18	1.34 ± 0.15<br>0.00-4.52	3.73 ± 0.17<br>1.38-7.34	27.18 ± 0.59<br>18.79-33.66	24.78 ± 0.60<br>16.64-31.37	1.74 ± 0.11<br>0.59-3.83	2.93 ± 0.07<br>2.05-3.73	1.53 ± 0.04<br>0.92-2.21
16		42	28.98 ± 0.61<br>17.65-39.20	2.73 ± 0.08<br>1.95-4.39	1.88 ± 0.15<br>0.00-3.78	4.59 ± 0.17<br>2.53-8.30	27.09 ± 0.58<br>16.71-35.82	24.39 ± 0.54<br>14.69-31.22	1.99 ± 0.12<br>0.36-3.92	2.81 ± 0.13<br>1.73-6.85	1.48 ± 0.05<br>0.92-2.62
20		42	28.72 ± 0.58<br>22.53-35.72	2.97 ± 0.08<br>1.96-4.55	2.34 ± 0.11<br>0.81-3.82	4.50 ± 0.14<br>2.26-5.90	26.37 ± 0.56<br>19.91-33.76	24.22 ± 0.56<br>17.55-30.71	1.35 ± 0.12<br>0.00-3.75	3.07 ± 0.09<br>2.18-4.77	1.76 ± 0.06<br>1.27-2.89
24		44	27.61 ± 0.54<br>17.60-33.18	2.99 ± 0.06<br>2.18-3.90	2.22 ± 0.13<br>0.51-4.37	4.40 ± 0.16<br>2.62-7.44	25.39 ± 0.55<br>16.63-31.68	23.20 ± 0.52<br>14.83-29.22	1.57 ± 0.12<br>0.16-3.91	3.07 ± 0.09<br>1.62-4.14	1.54 ± 0.05<br>0.92-2.17

T. simiae TV008 *PFR1::YFP*

Time	N	L	W	KPost	NPost	KAnt	NAnt	KNuc	NL	NW
0	65	44.38 ± 0.60<br>33.20-60.05	1.75 ± 0.03<br>1.30-2.53	11.89 ± 0.35<br>5.09-21.61	16.58 ± 0.37<br>6.66-25.77	32.48 ± 0.40<br>27.42-40.54	27.79 ± 0.41<br>21.65-36.16	3.98 ± 0.14<br>0.49-6.19	3.61 ± 0.08<br>2.34-5.12	1.16 ± 0.02<br>0.83-1.69
4	38	43.12 ± 0.95<br>34.63-53.74	2.03 ± 0.06<br>1.04-2.84	8.91 ± 0.63<br>1.31-16.43	12.59 ± 0.73<br>3.23-22.17	34.21 ± 0.55<br>25.14-41.22	30.54 ± 0.57<br>22.37-38.55	2.90 ± 0.17<br>0.36-5.46	3.79 ± 0.13<br>2.30-6.34	1.21 ± 0.03<br>0.92-1.85
8	26	37.72 ± 1.00<br>27.60-46.35	2.21 ± 0.06<br>1.62-3.07	5.63 ± 0.63<br>1.04-12.73	8.71 ± 0.67<br>4.41-16.51	32.09 ± 0.66<br>21.86-38.98	29.01 ± 0.66<br>19.07-34.28	2.54 ± 0.16<br>1.31-4.21	3.31 ± 0.09<br>2.48-4.19	1.24 ± 0.03<br>0.87-1.54
12	14	33.40 ± 1.27<br>23.57-31.34	2.01 ± 0.1<br>1.47-2.90	3.30 ± 0.49<br>0.67-7.15	5.99 ± 0.57<br>3.12-9.42	30.10 ± 1.46<br>17.98-40.68	27.42 ± 1.49<br>14.37-36.89	2.11 ± 0.26<br>0.83-2.11	3.22 ± 0.09<br>2.76-3.89	0.99 ± 0.05<br>0.73-1.40
24	14	35.09 ± 1.08<br>28.56-43.41	2.13 ± 0.10<br>1.67-3.13	4.20 ± 0.41<br>1.73-6.49	6.82 ± 0.49<br>3.06-8.74	30.89 ± 0.88<br>25.80-39.27	28.27± 0.84<br>23.95-35.44	2.02 ± 0.21<br>0.82-3.65	3.69 ± 0.15<br>2.48-4.65	1.35 ± 0.06<br>1.03-1.85
30	16	34.38 ± 1.34<br>27.99-45.06	2.13 ± 0.08<br>1.67-2.77	5.70 ± 0.36<br>3.65-9.19	8.07 ± 0.32<br>5.46-11.08	28.68 ± 1.21<br>21.84-38.02	26.31 ± 1.17<br>20.02-35.35	1.60 ± 0.22<br>0.00-3.65	3.31 ± 0.12<br>2.32-4.09	1.18 ± 0.05<br>0.81-1.54
36	4	33.56 ± 5.37<br>26.22-49.26	2.26 ± 0.37<br>1.67-3.29	5.61 ± 1.04<br>3.99-8.54	8.41 ± 1.07<br>6.47-10.98	27.95 ± 4.46<br>21.39-40.73	25.15 ± 4.41<br>19.76-38.28	2.17 ± 0.70<br>0.65-3.95	4.10 ± 0.32<br>3.33-4.84	1.21 ± 0.04<br>1.15-1.30

Time	N	FL	FPost	FAnt
0	65	30.25 ± 0.50<br>22.25-39.26	14.31 ± 0.35<br>7.16-23.96	1.84 ± 0.19<br>0.00-6.91
4	38	31.16 ± 0.57<br>22.26-38.57	11.19 ± 0.66<br>2.29-18.99	1.56 ± 0.30<br>0.00-10.74
8	26	29.45 ± 0.68<br>21.66-34.89	7.98 ± 0.62<br>3.15-15.26	1.48 ± 0.29<br>0.00-4.46
12	14	28.56 ± 1.60<br>13.74-40.64	5.06 ± 0.63<br>1.89-9.24	0.71 ± 0.26<br>0.00-2.82
24	14	29.16 ± 0.88<br>25.60-36.91	6.44 ± 0.50<br>3.09-8.94	1.82 ± 0.31<br>0.00-4.25
30	16	26.65 ± 1.12<br>22.19-36.40	8.09 ± 0.38<br>5.96-12.12	1.25 ± 0.41<br>0.00-4.86
36	4	27.31 ± 4.52<br>20.30-40.14	8.04 ± 0.98<br>5.91-10.47	0.83 ± 0.30<br>0.00-1.39
