# Supplementary figures and images for: Development of the livestock pathogen Trypanosoma (Nannomonas) simiae in the tsetse fly with description of putative sexual stages from the proboscis
Source: Parasit Vectors. 2023 Jul 11;16:231. doi: 10.1186/s13071-023-05847-5 (PMC10337175; doi:10.1186/s13071-023-05847-5)

A

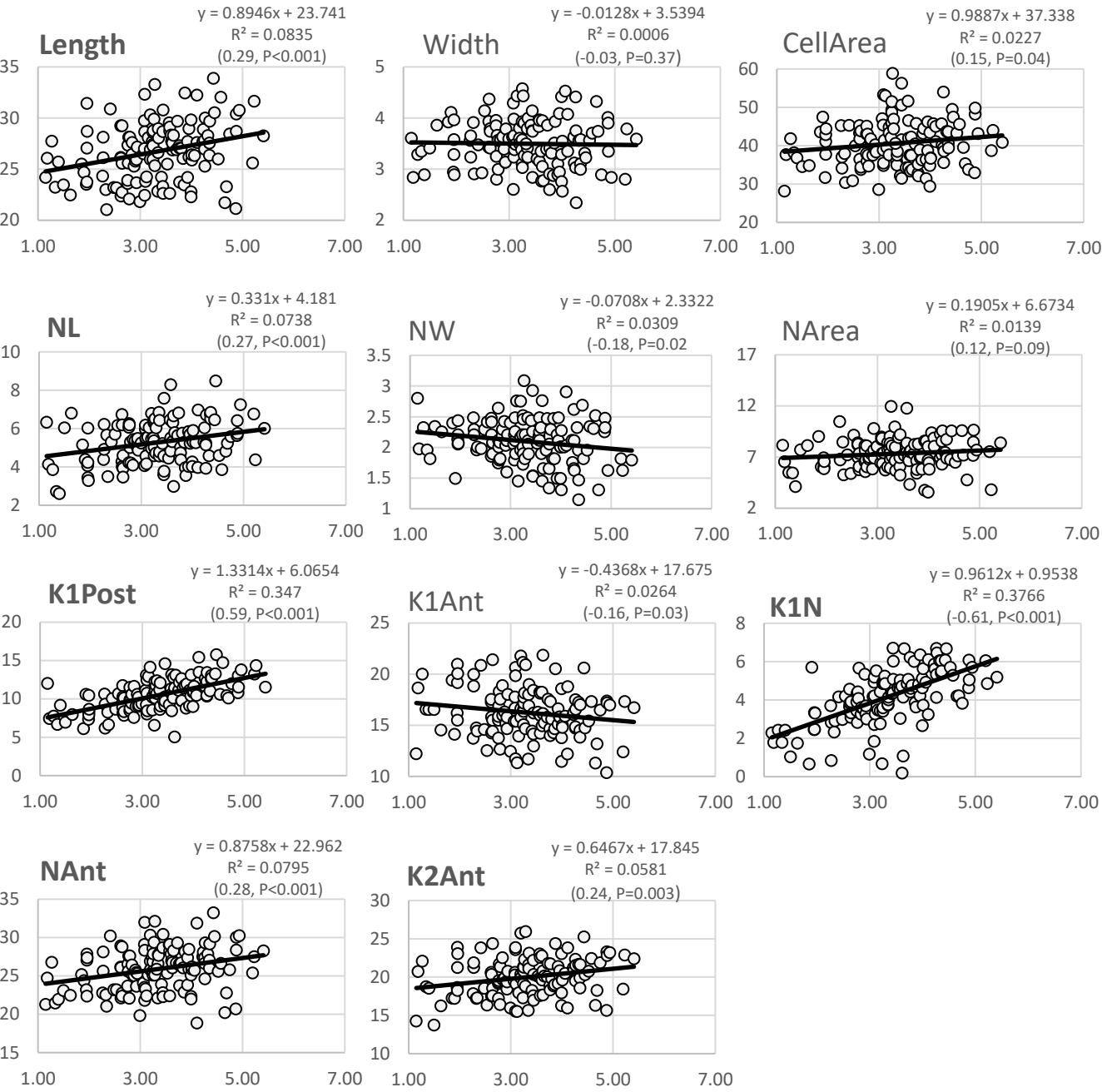

B

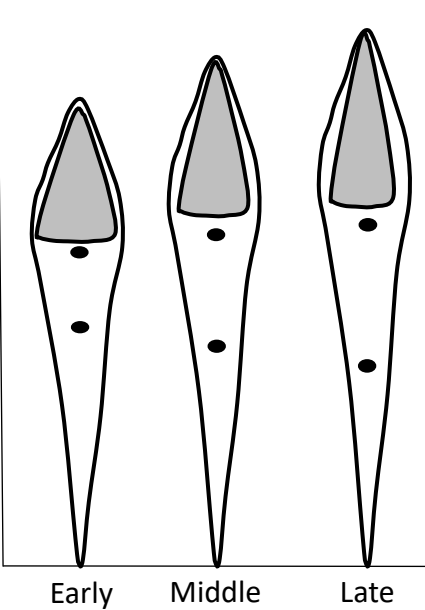

Supplement: Supplementary file 4 — Additional file 4: Fig. S1. Analysis of meiotic dividers. Correlations and diagram based on measurements of 129 2K1N meiotic dividers. A Correlations for 11 measured variables. In linear regression graphs, X-axis is K1-K2 distance (µm) and Y-axis is an individual morphometric (µm); above each graph is the R2 value, with the Pearson correlation coefficient and P value below in brackets. B Schematic of progression based on the increasing distance between the two kinetoplasts (K1-K2) and the 11 other morphometrics, some of which changed at the same time. K1 is closest to the cell anterior. [file 13071_2023_5847_MOESM4_ESM.pdf]

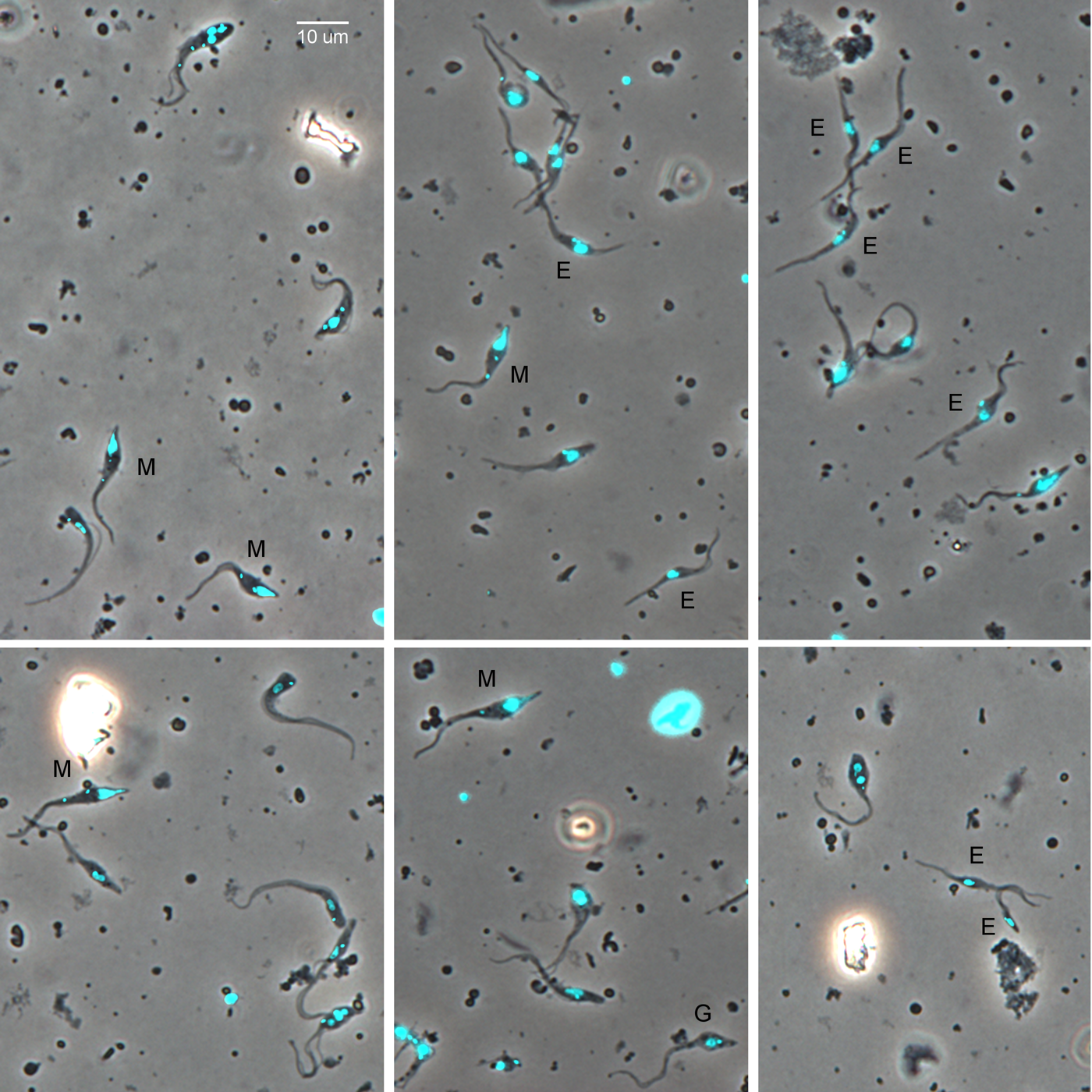

Supplement: Supplementary file 5 — Additional file 5: Fig. S2. Images of trypanosomes from the proboscis. These images of part microscope fields give a feel for the abundance of meiotic dividers (M) and gametes (G) among epimastigotes (E) and other cell types from the proboscis. Scale bar = 10 um. [file 13071_2023_5847_MOESM5_ESM.tif]
